# Do Different Types of Assets Have Differential Effects on Child Education? Evidence from Tanzania

**DOI:** 10.1016/j.worlddev.2018.04.006

**Published:** 2018-04-21

**Authors:** Kashi Kafle, Dean Jolliffe, Alex Winter-Nelson

**Affiliations:** a Research ad Impact Assessment Division, International fund for Agricultural Development (IFAD), Rome, Italy; b Development Research Group, World Bank, Washington, DC, USA; c Department of Agricultural and Consumer Economics, University of Illinois, Urbana, IL, USA

**Keywords:** I25, J22, D13, O12, LSMS-ISA, Tanzania, asset ownership, child education, highest grade completed, school performance

## Abstract

To assess the conventional view that assets uniformly improve childhood development through wealth effects, this paper tests whether different types of assets have different effects on child education. The analysis indicates that household durables and housing quality have the expected positive effects, but agricultural assets have adverse effects on highest grade completed and no effects on exam performance. Extending the standard agricultural-household model by explicitly including child labor, the study uses three waves of panel data from Tanzania to estimate the effects of household assets on child education. The analysis corrects for the endogeneity of assets and uses a Hausman-Taylor instrumental variable panel data estimator to identify the effects of time-invariant observables and more efficiently control for time-invariant unobservables. The negative effect of agricultural assets is more pronounced among rural children and children from farming households, presumably due to the higher opportunity cost of their schooling.

## Introduction

While poverty is typically defined by whether someone has sufficient daily income or consumption to meet basic needs, many development organizations view wealth creation through asset ownership as ultimately the pathway out of poverty. For example, a core element of a poverty-reduction program in Bangladesh, run by BRAC (the largest nongovernmental development agency in the world) and replicated in over 20 countries, is an asset transfer program targeted to poor households. Banerjee et al. (2015) present evidence from randomized controlled trials from several countries that had asset transfer programs similar to the BRAC program as the core element, and show that the effects on poverty reduction are significant and long-lasting.^1^

Owning more assets increases household wealth, and greater wealth can improve well-being in many different ways. One path is through increased investment in human capital, which can break cycles of poverty and help to extract people from chronic poverty. A large body of evidence has established that having more physical assets results in greater investment in children’s education, particularly in richer countries (Chowa et al., 2013; Conley, 2001; Deng et al., 2014; Elliott et al., 2011; Elliott and Sherraden, 2013; Huang, 2011, 2013; Kim and Sherraden, 2011; Loke, 2013; Shanks, 2007; Zhan and Sherraden, 2003).^2^ There is also a fairly extensive body of research on the ‘asset-child education’ relationship in developing countries. Deng et al. (2014) and Filmer and Pritchett (2001) construct a measure of wealth based on assets and examine child education outcomes; others, like Chowa et al. (2013) and Cockburn and Dostie (2007), construct measures of asset ownership and examine education outcomes. Chowa et al. (2013) find that Ghanaian children in households that own at least one of five assets — TV, refrigerator, electric iron, electric or gas stove, and kerosene — outperformed the control group in English test scores. Similarly, Filmer and Pritchett (2001) construct an asset-based wealth indicator in India and find a rich-poor gap of more than 30 percent in school enrollment rates.

A common aspect of the studies establishing a positive link between owning more assets and better child-education outcomes is the implicit assumption that the type of asset does not affect this relationship. Most studies either monetize or count asset holdings, converting all assets into a singular wealth scalar, and find a positive relationship between wealth and child education. One question we explore in this paper is whether an undifferentiated view of assets ignores the potential for different types of assets to have varying effects on child education. More specifically, we explore whether agricultural assets discourage education investment, possibly by increasing the returns to child labor, while other assets (e.g., electricity or bicycle) could contribute to child education by heightening the returns to schooling or raising the efficiency of time spent studying.

If different types of assets have differential effects, there may be significant scope to improve the design of asset transfer and public investment programs. Such programs usually transfer income-generating assets, such as livestock (Jodlowski et al., 2016; Kafle et al., 2016; Rawlins et al., 2014); agricultural inputs (Denning et al., 2009); and other in-kind physical assets (Banerjee et al., 2015; Muralidharan and Prakash, 2013). Although physical asset transfers may in the short run provide a practical approach for programs to improve livelihoods, some assets could influence the returns to child labor in ways that discourage investment in formal education and thus hurt longer-term economic development.

Our contribution to the literature is twofold: (1) We establish a theoretical relationship between different types of assets and child education under both perfect and imperfect labor market conditions. (2) In the empirical analysis, we provide evidence that different types of assets have differential effects on child education. Specifically, we show that household durables and housing quality indicators have the expected positive effects but agricultural assets affect child education negatively. We also demonstrate that the negative effect of agricultural assets is more pronounced among rural children and children of crop producers, which we argue stems from the higher opportunity cost of their schooling.

In what follows, section 2 sets out our theoretical model, which builds on the Basu et al. (2010) model of child labor and landholding, which in turn adopts the agricultural household model of Singh et al., (1986). Our primary extension is to introduce an education production function that constrains the household’s utility maximization problem. In section 3, we describe our data — three waves of the Tanzania National Panel Survey (NPS)^3^ -- and empirical model. In section 4, we discuss both the descriptive and the empirical results. Rather than assuming undifferentiated effects of assets, we categorize assets into three types — household durables, agricultural, and housing quality — and estimate how each type affects children’s educational outcomes. The findings demonstrate that different types of assets have differential effects. Section 5 discusses the policy implications of our conclusions.

## 2 Theoretical model and results

While the hypothesis that different types of assets can have differential effects on child education is intuitively appealing and empirically testable, there has been significantly less consideration of the theory of this relationship. A handful of studies model the asset-education relationship using variants of the agricultural household model, but they do not differentiate between types of assets. Whenever the expected return to schooling is less than the return to child labor, providing households with more assets can have adverse effects on child education (Cockburn and Dostie, 2007). That child labor adversely affects child education is a common finding (Basu et al., 2010; Haile and Haile, 2012). Basu et al. (2010) develop this further and show that when labor markets are complete, an increase in household wealth (measured by land holdings) decreases child labor and improves child education. However, when labor markets are incomplete, the effect of land holdings on child labor (hence child education) is ambiguous: it depends on how the underlying utility and production functions are specified.

The conceptual framework considered here develops intuitively appealing theoretical and empirical bases for expecting different assets to have differential effects on child education. We explicitly assume that child labor adversely affects children’s educational outcomes and examine the asset-child labor relationship, drawing from the agricultural household models described in Singh et al. (1986). Starting from the basic structure as described in Basu et al. (2010), we introduce an education production function that constrains the household’s utility maximization. We consider the cases of both perfect-labor markets and missing-labor markets. Because our primary interest is in the interactions between assets and human capital investments in education, we focus on scenarios where the household is constrained by an education production function, but for the sake of completeness, we also consider scenarios where it is not. Table 1 presents all four permutations — perfect and missing labor market, with and without an education production function.

**Table 1:** Effects of exogenous increase in assets and income on child labor and household consumption

	Perfect labor market				No labor market			
	Case 1		Case 2		Case 3		Case 4	
	*l*	*c*	*l*	*c*	*l*	*c*	*l*	*c*
Agricultural assets (K)	-ve	+ve	-ve	+ve	±	+ve	±	+ve
Assets specific to child education (A)	.	.	+	-ve	.	.	+ve	-ve
Income (y)	-ve	+ve	-ve	+ve	-ve	+ve	-ve	+ve
Education production function (q)	No		Yes		No		Yes	

*Notes. l* indicates child labor, and *c* indicates household consumption. Similarly, -ve, +ve, and ±, indicate negative, positive, and ambiguous effects of assets or income, respectively, on child labor and household consumption.

Consider an economy where each household has one adult and one child. The adult always prefers to work and takes no leisure. The child either works or goes to school but takes no leisure. Suppose each household has the following utility function:

(1) $$u=u\left(c,l\right)$$

where *c* is total consumption and $l\in \left[0,1\right]$ is child labor hours; 0 indicates no child labor, and 1 indicates no school/study hours. Since the adult always prefers to work, the total labor supply of the household is always *1+l.* The aggregate consumption good *c* increases utility but labor accrues disutility. We assume that the utility function is smooth and quasi-concave and the following relationship holds: *u_c_* > *0,u_cc_ ≥0,u_l_*< 0, and *u_u_* < 0.^4^ Similarly, we assume that the cross-marginal utilities are negative: *u_cl_, u_lc_ <* 0. Each household faces a budget constraint, is engaged in household production activity, and owns both agricultural assets (*K*) and nonagricultural assets *(A)*. If a household has a child who attends school, the household also faces an education production function and is liable for the cost of schooling, *p_q_*.

### The perfect labor market case

Assuming perfect labor markets, a household can supply labor to off-farm activities and hire outside labor to work on its farm at a market wage rate, *w*. Following Basu et al. (2010), we assume that both adults and children earn exactly the same wage. Suppose each household faces a production function Q(L,K), and an education production function, *q*(s,A,θ),^5^ where *L* is total labor used in household production, *K* is the household’s agricultural assets, *s=1-l* is total school/study hours, *A* is the household’s non-agricultural assets, which may directly affect child education, and 0 denotes ‘other factors’ that affect child education. For simplicity, we suppress 0 and assume that the education production function is linear on school hours, i.e., *q(s,A) = s + q(A)*. Because the household production function is quasi-concave, *Q_L_, Q_K_ >* 0; *Q_LL_ <* 0; *Q_LK_ >* 0. We assume *q_s_, q_A_* >0 and *q_ss_* = 0. The household’s problem is:

(2) $$Q=Q\left(L,K\right)\;q=q\left(s,A\right)\;\mathrm{and}\;c+p_{q}q=Q+y+w\left(H-L\right)$$

where *Q* is output produced, *q* is children’s educational outcomes, is the unit cost of child education, *y* is non-labor income, and *H* = 1+ *l* is the household’s total labor supply. A household supplies labor off-farm if *H > L* and hires labor from outside if *H < L*. Since the labor market is well- functioning and the household can hire labor in or out as needed, the production decision is separable from the consumption decision. A household that possesses *K* units of agricultural assets can earn a profit of *π(w, K)*. Therefore, *c* + *p_q_q* = *π(w, K)* + *wH* + y. The household’s problem simplifies to

(3) $$u\left(c,l\right)-\lambda \left[c+p_{q}q-\pi \left(w,k\right)-w\left(1+l\right)-y\right]$$

Rearranging the first-order conditions from equation (3) gives us the following expressions:

(i) $$\frac{u_{l}}{u_{c}}\equiv Z=-\left(p_{q}+w\right)$$

(ii) $$c+p_{q}q=\pi \left(w,K\right)+w\left(1+l\right)+y$$

Totally differentiating these expressions with respect to *K* and solving the resulting equations,

we get

$$\frac{\delta l}{\delta K}=-\frac{z_{c}\pi_{K}}{z_{c}\left(p_{q}+w\right)+z_{l}}\;\mathrm{and}\;\frac{\delta c}{\delta K}=\frac{z_{l}\pi_{K}}{z_{c}\left(p_{q}+w\right)+z_{l}}$$

By assumption, *π_K_ >0, q_s_* > 0, and we can demonstrate that *z_c_* <0, *z_l_*; < 0.^6^ Therefore, when the labor market is perfect, a household that accumulates agricultural assets decreases child labor, i.e.,$\frac{\delta l}{\delta k}<0$, but increases household consumption, i.e.,$\frac{\delta c}{\delta k}>0$. Similarly, differentiating expressions i.) and ii.) with respect to income *y* gives us the following conditions:

$$\frac{\delta l}{\delta y}=-\;\frac{z_{c}}{z_{c}w+z_{l}}<0\;\mathrm{and}\;\frac{\delta c}{\delta y}=\frac{z_{l}}{z_{c}w+z_{l}}>0$$

This indicates that an exogenous increase in income or assets unambiguously reduces child labor and increases consumption when the labor market is perfect. Differentiating conditions i) and ii) with respect to nonagricultural assets, A, shows that an exogenous increase in education-specific assets may reduce household consumption $\left(\frac{\delta c}{\delta A}<0\right)$ and increase child labor hours $\left(\frac{\delta l}{\delta A}>0\right)$.

The results imply that, when the labor market functions perfectly, the income effect on child labor is always negative, but the effect of owning assets depends on the type of assets. Since assets are likely to affect household income, the net effect of an increase in assets is ambiguous, and the ambiguity gets more complicated where there is no labor market.

### 2.2 The missing labor market case

In this case a household’s consumption decisions are non-separable from production decisions. No outside labor is hired and no household labor is supplied to off-farm activities. Since there is no market wage, the household’s problem in (2) can be modified as

(4) $$Q=Q\left(L,K\right),$$

$$q=q\left(s,A\right)\;\mathrm{and}\;c+p_{q}q=Q+y$$

Because of non-separability, the household’s problem simplifies to

(5) $$u\left(c,l\right)-\lambda \left[c+p_{q}q\left(s,A\right)-Q\left(L,K\right)-y\right]$$

Solving equation (5) gives us the following first-order conditions (FOCs)

$$\frac{u_{l}}{u_{c}}\equiv Z=-\left(p_{q}+Q_{L}\right)$$

$$c+p_{q}q=Q+y$$

Differentiating the FOCs with respect to agricultural assets, *K*, we get

$$\frac{\delta l}{\delta K}=-\frac{Q_{K}z_{c}+Q_{LK}}{\beta }$$

where $\beta =z_{l}+Q_{LL}+z_{c}\left(p_{q}+Q_{LL}\right)<0$.

The denominator *β* is always negative, but the sign of the numerator depends on the sign of the expression *Q_K_Z_c_* + *Q_LK_*.Since we assume *Q_K_, Q_LK_* > 0 and *z_c_* < 0, this implies that the effect of agricultural assets on child labor is ambiguous; whether it increases or decreases child labor depends on the magnitude of the change in the marginal product of labor caused by additional agricultural assets. The ambiguity is further complicated by the fact that assets contribute to household income, and the income effect on child labor may work in a different direction than the direct effects of assets. To understand the income effect, we differentiate the FOCs with respect to nonlabor income *y*, and get $\frac{\delta l}{\delta y}=-\frac{z_{c}}{\beta }<0$. An exogenous increase in income decreases child labor, unambiguously. Similarly, the income effect on household consumption is always positive: $\frac{\delta c}{\delta y}=\frac{z_{l}+Q_{LL}}{\beta }>0$

Table 1 summarizes our theoretical results. The results in Cases 1 and 3 essentially replicate those of Basu et al. (2010) and Cockburn and Dostie (2007) except that we use agricultural assets in general rather than just land ownership. Cases 2 and 4 are novel and more realistic in that they consider both household and education production functions and explicitly model the cost of education. Overall, the results imply that the effects of an exogenous increase in assets and income are clearly discernible when the labor market is perfect. When no labor market exists and households have to make production and consumption decisions simultaneously, non-labor income and education- specific assets still have clearly discernible effects on child labor and consumption, but the effects of assets used in agricultural production are harder to understand because they are more complicated.

## 3 Method and Data

The initial focus of our empirical analysis is to unpack the ambiguous effect of assets on child education. Our empirical findings are consistent with the theoretical results in that household income always has a positive effect on children’s educational outcomes and the effect of assets depends on the type of assets.

### 3.1 Econometric model

Our empirical approach assumes labor markets are incomplete and that household decisions are nonseparable. As described in section 2.1, children’s educational performance (*q*) is determined by school hours (*s*), nonagricultural assets *(A),* and other factors (θ). Assume that among the other factors are parental characteristics, household income *(I),* and child’s individual ability (*C_u_*); and that school hours depend on agricultural assets *(K)* and household income. Parental characteristics consist of observed characters, such as education (*P_e_*), and unobserved characters, such as ability (*P*_u_). Conceptually, child education is a function of parental characteristics, child ability, assets, and income:

(6) $$q=P_{e}+P_{u}+C_{u}+A+K+I+error$$

We know that certain parental characteristics like hereditary traits and other abilities are transmitted directly to their children, i.e., *C_u_ = f (P_u_)* + *error*. This implies that children’s educational outcomes can be predicted by observed parental characteristics, child’s ability, assets, and income, i.e., *q = P_e_ + Č_u_ + A + K + I + error*. Note that *Č_u_ = C_u_ + f* ^-1^(*C_u_*) is unobserved ability that is both inherited from parents and specific to the individual child. Since parental ability is correlated with parental education and household assets, unobserved child ability (*C_u_*) is also correlated with both, i.e., *corr(C_u_,P_e_)*≠0 and *corr(C_u_,A)*≠ 0.

The fact that observed and unobserved variables are correlated and affect child education raises the problem of endogeneity. This is a concern which the existing literature has not yet addressed (Elliott et al., 2011; Lerman and McKernan, 2013). Assuming that these unobserved characteristics are time-invariant, we use panel data to address the endogeneity problem empirically. We estimate the following model with panel data:

(7) $$q_{it}=x_{1it}\alpha +x_{2it}\beta +z_{1i}\theta +z_{2i}\gamma +u_{i}+\varepsilon_{it}$$

where *i* indicates individual and *t* indicates time (survey round). Thus, *q_it_* is child *i’s* education outcome at time *t*; *x*_1*it*_ is a vector of time-varying exogenous variables, such as age and household size; *x*_2*it*_ is a vector of time-varying endogenous variables, such as assets; is a vector of time-invariant exogenous variables, such as gender and age started school; *z*_2*i*_ is a vector of time-invariant endogenous variables, such as maximum parent’s education; *u_i_* is a time-invariant individual effect that consists of unobserved individual abilities correlated with both asset ownership and parental education; and *ε_it_* is an idiosyncratic error term. Equation (7) provides the structure required for an instrumental variable estimator (hereafter referred to as HTIV), as proposed by Hausman and Taylor (1981), to address the endogeneity problem.

The HTIV model relies on instruments that come from within the model: *z*_1*i*_ serves as an instrument for itself; the within transformations x1it-x1i and x2it-x2i serve as valid instruments for *x*_1*it*_ and *x*_2*it*_, respectively; and the between transformation x1i serves as a valid instrument for *z*_2*i*_. Conditions (i) and (ii) are both necessary and sufficient conditions for the HTIV estimator to produce unbiased estimates:

$$E\left(u_{i}|x_{2it}\right)\neq 0,\;E\left(u_{i}|z_{2i}\right)\neq 0$$

$$E\left(u_{i}|x_{1it}\right)=0,\;E\left(u_{i}|z_{1i}\right)=0\;\mathrm{and}\;E\left(\varepsilon_{it}|x_{1it},z_{1i},z_{2i}\right)=0$$

This analysis assumes that the idiosyncratic error term is not correlated with any explanatory variables but that the unobserved specific effect is correlated with both asset indexes (*x*_2*it*_) and parental education (*z*_2*i*_).

Estimating equation (7) with the random effects model yields inconsistent estimates because the ‘zero correlation’ assumption is clearly violated. The fixed effects model and the HTIV method^7^both yield consistent estimates, but the HTIV approach is more efficient and can also estimate coefficient estimates on time-constant variables (Baltagi et al., 2003; Hausman and Taylor, 1981). Efficiency gain is particularly important for our analysis because the data come from a comprehensive nationally representative survey that is likely to have suffered from unforeseen measurement errors. Also, we wanted to estimate the effects of time-constant variables like parent’s education and gender. For these reasons, our preferred method is HTIV. However, for comparison purposes, we provide results from three different estimators: random effects, fixed effects, and HTIV.

### 3.2 Outcome variables

**Figure 1:**
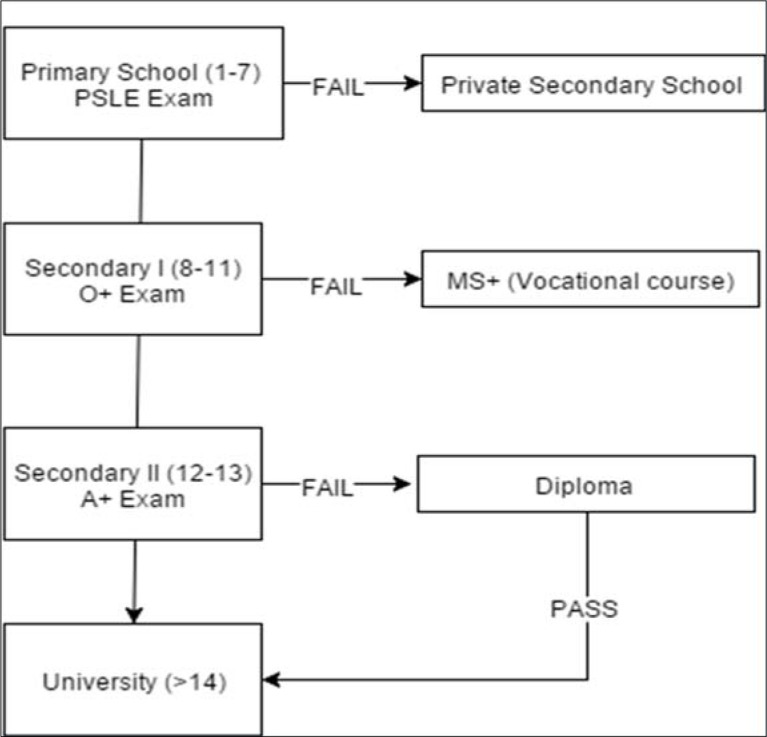
Educational system in Tanzania

This analysis assesses children’s educational outcomes in the context of progression through the Tanzanian school system, represented in Figure 1. Tanzania follows a 2-7-4-2-3+ model of education that starts with 2 years of preprimary school followed by 7 years of primary school, which ends with a national examination, the primary school leaving exam (PSLE), at the end of the 7^th^ grade (MoEVT, 2014). A pass score on the PSLE is required to proceed to government secondary school. Those who fail can either retake the exam or enroll in private secondary school.

The first tier of secondary school ends after 11^th^ grade with another national examination, the Form IV exam (the FIVE), or the O+ exam. Students passing the FIVE can move up to the second tier of secondary school; those who fail can either retake the exam or enroll in vocational courses (MS+). The second tier of secondary school ends after grade 13 with yet another national examination, the Form VI exam (the A+ exam). Students passing the A+ exam can go directly to university, another 3+ years of formal education; those who fail must pass a diploma course before they can attend university. Secondary school through the A+ exam in Tanzania is equivalent to high school in the United States.

Based on the school system, outcome variables for this analysis were chosen to estimate the effects of assets on both school enrollment and performance. The variables are: highest grade completed, PSLE ratio — the proportion of children who passed the PSLE over the number of children in the same household who are eligible for the PSLE (ages 6—18); and the FIVE ratio — the proportion of adolescents who passed the FIVE over the number in the same household who are eligible for it (ages 16-24). The highest grade completed is a count variable ranging from 1 to 25. A grade of 25 marks the earning of an advanced university degree (e.g., a PhD in the United States). For the highest grade completed, the analysis covers only individuals aged 6-18 in the first round. Individuals who have never attended school or attended only informal schools are excluded because neither school outcome nor school-related explanatory variables are available for them. Therefore, because our results are conditional on school attendance, inferences from the results should be drawn with caution.

While the highest grade completed is measured for individuals, the PSLE and FIVE ratios are aggregated to the household level because there is little to no variation in individual outcomes over time. The test scores are binary variables, pass or fail, and individuals who pass the exam once never retake the same exam. In our sample, about 65 percent of students pass the PSLE in the first attempt and the retake rate is very low; only about 13 percent of students unsuccessful in the first attempt succeed in the second. We have a little variation to work with because 95 percent of children have either 1 or 0 throughout and only 5 percent see their scores change over time from 0 to 1. The pattern for FIVE scores is similar. Aggregating the individual-level test performance into a household level variable results in variation that allows us to distinguish between households where all children are passing exams from households with varying degrees of less success. While this aggregation allows us to examine test performance, using the household-level ratio prevents direct inference about individual performance in the PSLE and FIVE.

### 3.3 Asset variables

Assets are broadly defined here as household durables, housing quality characteristics, and agricultural assets. The three groups give a total of 59 asset variables (Appendix Table A1). There are 23 household durables (tools and equipment used in the household), from televisions and cellphones to bicycles. There are 14 housing quality characteristics, such as floor, roof, and wall materials; number of rooms; and access to electricity, safe drinking water, and toilet facilities. Among the 22 agricultural assets are farm tools and equipment, livestock, and livestock-related assets.

**Table A1:** Pooled scoring factors and baseline summary statistics of asset variable

Household durables	Mean	Scoring factors	Agricultural assets	Mean	Scoring factors	Housing quality characteristics	Mean	Scoring factors
Radios	0.79	0.15	Hoes	2.27	0.03	Own dwelling (1=Yes 0=No)	0.78	-0.26
TVs	0.24	0.36	Spraying machines	0.04	0.10	Rent dwelling (1=Yes 0=No)	0.14	0.26
Telephones	0.02	0.12	Water pumps	0.02	0.13	House wall (1=cement/concrete, 0=else)	0.28	0.34
Mobile phone	1.11	0.30	Reapers	0.00	0.31	House roof (1=metal sheets 0=else)	0.66	0.25
Refrigerators	0.14	0.33	Tractors	0.00	0.31	House floor (1=concrete/cement/tiles 0=else)	0.43	0.36
Sewing machines	0.13	0.19	Trailers	0.00	0.31	Number of rooms (=1 if 3 or more 0=else)	0.55	0.01
Video/DVDs	0.19	0.26	Ploughs	0.07	0.08	Safe water (1=boiled/bottled/treated 0=else)	0.33	0.17
Computers	0.07	0.05	Harrows	0.00	0.31	Water source (1=protected, 0=open source)	0.53	0.24
Irons	0.36	0.29	Milking machines	0.00	0.40	Water hauling time(1=less than average, 0 else)	0.62	0.00
Electric/gas stoves	0.08	0.26	Harvesters/threshers	0.00	0.40	Access to toilet (1=Yes 0=No)	0.90	0.04
Other Stoves	0.69	0.24	Hand miller	0.01	0.22	Toilet type (1=modern, 0=Vault/Pit)	0.13	0.26
Water heaters	0.04	0.23	Coffee pulper	0.00	0.25	Electricity (1=Yes 0=No)	0.23	0.37
Cassette players	0.02	0.07	Fertilizer distributors	0.00	0.38	Fuel(1=electricity/gas/generator/solar,0=else)	0.24	0.37
Music systems	0.03	0.12	Livestock	3.84	0.03	Cooking fuel (1=firewood 0=else)	0.71	-0.36
Cars	0.05	0.23	Poultries	5.81	0.01			
Motor cycles	0.05	0.14	Donkeys	0.06	0.04			
Carts	0.54	0.05	Plots	1.61	0.02			
Bicycles	0.02	0.09	Outboard engines	0.03	0.03			
Wheel barrows	0.03	0.07	Land owned (1=Yes, 0=No)	0.64	0.01			
Boats/canoes	0.01	0.02	Land rented (1=Yes)	0.06	0.00			
Houses	1.17	-0.03	Land shared (1=Yes)	0.01	0.00			
Fan/ACs	0.23	0.30	0.13	0.00				
Dish antennas	0.13	0.26						
*Observations*	3082	3082		3082	3082		3082	3082

*Notes*. All asset variables are in count, unless otherwise indicated. Asset indexes calculated by using binary indicators of asset ownership are not qualitatively different from the indexes resulting from count variables. Scoring factor is the weight that is used to calculate the first principal component. The first component explains 26 percent of the variance in durable assets

Because the list of assets is particularly extensive for each category (i.e. agricultural, household quality and household durables), and *a priori* we have no model of which combination of assets matters, we use principal component analysis (PCA) to assign weights to each asset based on their relative contribution to total variance (for each category). Following Filmer and Pritchett (2001), we interpret the first principal component as a proxy for socioeconomic status in part because it captures the largest variation in assets (see also 2001; Filmer and Scott, 2008; McKenzie, 2005; Vyas and Kumaranayake, 2006). Since this analysis uses longitudinal data, we need PCA weights for each wave. It would be possible to use period-specific weighting factors, but allowing weights to change over time produces asset indexes that are not comparable. To address this issue, we pool the waves to produce weighting factors for each asset that are constant over time; as has been done in related literature on using PCA with panel data (see for examples Harttgen et al., 2013; Booysen et al., 2008; Sahn and Stifel, 2003). Appendix Table A1 shows the weighting factors for each asset.

Other variables included in the analysis as controls are at the levels of both individuals (age, sex, age started school, and number of siblings) and households (age, sex, and marital status of household head and total consumption expenditure). Other controls are maximum parent’s education, binary indicators for school in local community, rural vs urban residence, economic shock in the last 12 months, and household access to credit and saving facilities.

### 3.4 Data

We use the data from the National Panel Survey (NPS) of Tanzania. The NPS is a nationally representative survey conducted by the National Bureau of Statistics of Tanzania in collaboration with the World Bank Living Standards Measurement Study — Integrated Surveys on Agriculture (LSMS- ISA). It tracks 3,265 baseline households and all of the split-offs of these households over time. Over the three waves, the attrition rate of households is 4.8 percent. Despite attrition, the sample size increases over time due to the design of following household members even as they separate from the original baseline household to form their own household. In the second wave of data collection, the number of panel households increases to 3,924; and in the third wave, to 5,010 households. The number of observations for individuals went up from 16,709 in the baseline to 20,599 in the second wave and 25,412 in the third. The attrition rate for individuals was 7.5 percent.

The NPS follows the same households and all household members age 15 or older (excluding live-in servants). We use a balanced sample from the three survey rounds. The full panel consists of 3,082 households and 14,552 individuals, but the sample size for this analysis varies with the outcome variable. For the highest grade completed, the panel is 4,112 children (from 2,241 households) who have once attended school and were aged 6-18 in the first wave. Similarly, for the PSLE variable, we used a panel of 3,101 households having at least one PSLE-eligible child aged 6-18, and for the FIVE variable, a panel of 2,696 households with at least one FIVE-eligible member aged 16-24.

## 4 Results

### 4.1 Summary statistics

Tables 2—4 present summary statistics. All point estimates are weighted to allow inferences to the population of either individuals or households, depending on the variable. Table 2 presents demographic characteristics of the sample in all three waves, disaggregated by individual characteristics, household characteristics, and characteristics of the household head. Tanzania has a very young population: in the NPS the average age is 21 years in the baseline, 23 in the second wave, and 24 in the third. For those who have attended school, the average age at start of school is 8, which is higher than the Sub-Saharan Africa average of 7 years. In all three waves, average household size is about 5, and about half of the individuals are children aged 6-18 years.

**Table 2:** Summary statistics of individual and household characteristics

Characteristics	Wave 1 (2008/09)	Wave 2 (2010/11)	Wave 3 (2012/13)	Observations
*Individual*				
Age	20.97 (0.150)	22.76 (0.150)	24.48 (0.144)	14552
Gender (1=male,0=female)	0.49 (0.004)	0.49 (0.004)	0.49 (0.004)	14552
Age started school†	8.01 (0.021)	8.01 (0.021)	8.01 (0.021)	9645
*Household*				
Expenditure, real (million TSZ)	2.50 (0.042)	2.91 (0.046)	3.84 (0.062)	3069
Maximum parent’s education‡	2.23 (0.023)	2.23 (0.023)	2.23 (0.023)	3064
Household size	5.01 (0.048)	5.27 (0.049)	5.26 (0.049)	3073
Number of children 6-18	2.79 (0.038)	2.89 (0.039)	2.84 (0.039)	3073
Shock in last 12 months (1=Yes,0=No)	0.53 (0.009)	0.41 (0.009)	0.36 (0.009)	3073
Rural	0.72 (0.008)	0.71 (0.008)	0.71 (0.008)	3069
School in village (1=Yes, 0=No)	0.89 (0.003)	0.94 (0.004)	0.96 (0.003)	3073
*Household Head*				
Age	45.3 (0.28)	47.1 (0.276)	48.7 (0.27)	3073
Gender (1=Male, 0= Female)	0.76 (0.008)	0.75 (0.008)	0.74 (0.008)	3073
Education level (grade)	2.27 (0.022)	2.27 (0.022)	2.27 (0.022)	3073
Marital status (1= Married, 0 else)	0.75 (0.008)	0.74 (0.008)	0.72 (0.008)	3073

† Number of observations of ‘age started school’ is much smaller than other variables because about 35 percent of the population has never attended school

‡ Maximum parent’s education is maximum education level of father or mother. It is coded as follows: 1= no education, 2= primary not finished, 3= primary, 4= secondary not finished, 5= secondary, and 6= higher than secondary.

*Notes*. Point estimates are population weighted means. Standard errors are in parentheses.

**Table 4:** Summary statistics of asset indexes across three waves

Asset indexes	Wave 1 (2008/09)	Wave 2 (2010/11)	Wave 3 (2012/13)
Aggregated asset index†	-0.227 (0.058)	0.020 (0.060)	0.207 (0.059)
Household durable index	-0.128 (0.043)	0.082 (0.044)	0.046 (0.043)
Agricultural asset index	0.067 (0.047)	0.025 (0.046)	-0.091 (0.011)
Housing quality index	-0.155 (0.038)	-0.028 (0.040)	0.182 (0.041)
Observations	3082	3082	3082

† The aggregated asset index consists of 59 variables, and three sub-indexes - household durable index, agricultural asset index, and housing quality index - consist 23, 22, and 14 variables, respectively.

*Notes*. Point estimates are population weighted means. Standard errors are in the parentheses. All asset indexes are constructed using the Principal Component Analysis (PCA) and the same loading factors obtained from the pooled data are used across three waves.

Parental and household head characteristics are important for the analysis because the effect of assets on child education mostly operates through parental decisions about child labor, schooling, and intra-household resource allocation. Parental education is measured by ‘maximum parent’s education’, the maximum education of father and mother. Since the vast majority of parents in the sample are not current students, we keep parental education constant across waves. On average, both parents and household heads have attended primary school, but about 20 percent are still illiterate. The other characteristics of household head we analyzed are age, gender, and marital status. Household heads are relatively young, averaging 45 years in baseline, 47 in the second wave, and about 49 in the third. More than 70 percent of household heads are married; the gender balance of headship is skewed to males, with only about 25 percent of households headed by females.

Apart from individual and household head characteristics, the effects of assets may differ by income level, rural or urban location, household response to transitory shocks, and access to a school in the local community. Apparently, even though more than 70 percent of households in the sample are rural, a strikingly large proportion (90 percent) have a primary or secondary school in the village. Access to primary and secondary schools in rural areas signals that the country has made a large investment in educating children (though the quality of education is not known). Descriptive results also indicate growing resilience over time: though 78 percent of households at some point experienced a negative economic shock, the poverty rates did not go up. Consistent with the reduction in national poverty rates (World Bank, 2015), average annual household consumption increased from T Sh2.5 million in the baseline to T Sh3.8 million in the third wave.

Table 3 summarizes children’s educational outcomes. Because educational outcomes are not available for children who have never attended school, both our summary statistics and the empirical results are conditional on attending school. We track the cohort of children aged 6-18 at baseline to estimate the effect of assets on ‘highest grade completed’. On average, children in the sample had completed 5^th^ grade at baseline, 7^th^ grade in the second wave, and 9^th^ grade in the third. As PSLE and FIVE data are not available for the first wave, we use only the PSLE and FIVE data from the second and third waves. Even though in both waves the passing rate for both tests is higher than 65 percent, only a small proportion of eligible children passed because most school-age children were not enrolled in school. Nevertheless, the proportion of school-age children passing the PSLE went up from 18 percent in 2010 to 23 percent in 2012. The pattern is similar for the FIVE.

**Table 3:** Summary statistics of child educational outcomes across three waves

Educational outcomes	Wave 1 (2008/09)	Wave 2 (2010/11)	Wave 3 (2012/13)	Observations
Highest grade completed	5.86 (0.050)	7.64 (0.053)	9.14 (0.054)	4112
PSLE pass ratio‡	-	0.18 (0.005)	0.23 (0.006)	3101
FIVE pass ratio‡	-	0.10 (0.005)	0.13 (0.006)	2696

‡ Primary school leaving exam (PSLE) and Form IV exam (FIVE) are national level examinations administered after 7^th^ and 11^th^ grades, respectively. The PSLE and FIVE ratios are the proportions of children passing the PSLE and FIVE tests to total children of ages 6-18 and 16-24, respectively. Because test scores data are not available for the first wave, both PSLE and FIVE ratios are presented for the second and third waves only.

*Notes*. Point estimates are population weighted means. Standard errors are in the parentheses.

Table 4 presents descriptive statistics for the asset indexes for all three waves. Since we calculate asset indexes at the household level, we assume that all children within a household have

equal access to household assets. The average value of the agricultural-asset index is approximately the same over each wave, suggesting no significant improvement over time in the total value of these assets. In contrast, both the index for household durables and housing quality increases over the span of the three waves.

### 4.2 Empirical results

We first examine the data to verify that having agricultural assets predicts child labor in agriculture. Pooling the data from the three waves, we estimate a probit regression of child labor on all three types of assets for various subsamples. We find that agricultural assets increase the likelihood of child labor among crop producers and rural households in general, but children are less likely to engage in any labor-generating activity if the family owns household durables and has housing quality assets (Appendix Table A2). This finding supports our assumption that effects of assets on child education operate through child labor. Next, we estimate the effect of asset-holding on children’s educational outcomes.

**Table A2:** Likelihood of child own-farm agricultural labor

	Model: Pooled Probit			
	Rural	Urban	Crop producers	Livestock keepers
Log(Total expenditure)	0.165^***^ (0.033)	0.053 (0.064)	0.123*** (0.031)	0.143^***^ (0.034)
Household durable index	-0.014 (0.016)	-0.011 (0.021)	-0.004 (0.015)	-0.025 (0.017)
Agricultural asset index	0.016^**^ (0.006)	-0.005 (0.004)	0.011^*^ (0.006)	0.008 (0.006)
Housing quality index	-0.222^***^ (0.018)	-0.192*** (0.026)	-0.168*** (0.017)	-0.133*** (0.018)
School in village (1=Yes, 0=No)	0.807^***^ (0.117)	0.404^***^ (0.105)	0.693^***^ (0.090)	0.549^***^ (0.105)
Max. parent’s education	-0.112*** (0.020)	-0.030 (0.030)	-0.100*** (0.018)	-0.079^***^ (0.018)
Gender (1=Male, 0=Female)	0.154*** (0.035)	0.218*** (0.069)	0.169*** (0.033)	0.152^***^ (0.035)
Head’s gender (1=Male, 0=Female)	0.005 (0.060)	-0.120 (0.133)	-0.065 (0.060)	-0.062 (0.063)
Age (years)	0.094^***^ (0.005)	0.049^***^ (0.010)	0.093^***^ (0.005)	0.096^***^ (0.005)
Age started school	-0.022 (0.013)	-0.045 (0.029)	-0.019 (0.013)	-0.015 (0.014)
Household size	-0.058^***^ (0.013)	-0.111*** (0.024)	-0.063^***^ (0.012)	-0.069^***^ (0.012)
*Observations*	8097	3897	8798	7219

*Notes*. Standard errors are in parentheses. Significance level: *p < .10, **p < .05, ***p < .01. Dependent variable is child labor in agriculture (1= yes, 0 = no) and the results are obtained from pooled probit model.

#### 4.2.1 Effects of assets on highest grade completed

We estimate the effects of assets on highest grade completed using equation (7) for two different model specifications with three different panel estimators: random effects, fixed effects, and HTIV. Both specifications are the same except for the treatment of asset variables. The first specification in Table 5 does not allow for analysis of differential effects of assets because it aggregates all assets into the same index, but the second specification covers all three disaggregated asset indexes (Table 6). Results in Table 7 also come from the second specification, estimated with our preferred HTIV model for various subsamples. In all regressions, standard errors are clustered at the household level. Tables are structured so that results in the first column are obtained from the random effects estimator, which is inconsistent under conditions (i) and (ii) shown section 3.1. Under the same conditions, results in the second and third columns are consistent because they are obtained from the fixed effects and the HTIV estimators; results in the third column are our preferred results because the HTIV estimator is consistent *and* more efficient than the fixed-effects estimator.

**Table 5:** Effects of aggregated asset index on highest grade completed, children aged 6-18

	Dep. variable: Highest grade completed		
	RE	FE	HTIV
Log(Total expenditure)	0.263*** (0.035)	0.152*** (0.040)	0.164*** (0.034)
Aggregated Asset index	0.098*** (0.011)	0.038** (0.016)	0.051*** (0.012)
School in village (1=Yes,0=No)	0.145** (0.063)	0.239^***^ (0.086)	0.248^***^ (0.073)
Max. parent’s education	0.224^***^ (0.025)	-	0.843^***^ (0.077)
Gender (1=Male, 0=Female)	-0.275^***^ (0.053)	-	-0.293^***^ (0.060)
Head’s gender (1=Male, 0=Female)	-0.229^***^ (0.076)	-0.289^**^ (0.114)	-0.046 (0.056)
Age (Years)	0.810^***^ (0.008)	0.814^***^ (0.010)	0.814^***^ (0.006)
Age started school	-0.463^***^ (0.025)	-	-0.431^***^ (0.024)
Household size	-0.080^***^ (0.014)	-0.079^***^ (0.017)	-0.074^***^ (0.013)
Observations	11992	11992	11992

*Notes*. Standard errors are in parentheses. Significance level:

* *p* < .10

** *p* < .05

*** *p* < .01. Results are based on panel of children who have attended school and were 6 to 18 years old in 2008. Results are presented for key variables only, estimated model includes additional variables. RE, FE, and HTIV stand for Random Effects, Fixed Effects, and Hausman-Taylor Instrumental Variable estimators, respectively.

**Table 6:** Effects of different assets on highest grade completed, children aged 6-18

	Dep. variable: Highest grade completed		
	RE	FE	HTIV
Log(Total expenditure)	0.241*** (0.035)	0.146*** (0.040)	0.157^***^ (0.034)
Household durable index	0.073^***^ (0.014)	0.020 (0.018)	0.026^*^ (0.015)
Agricultural asset index	-0.009^***^ (0.003)	-0.012*** (0.004)	-0.013*** (0.004)
Housing quality index	0.096^***^ (0.017)	0.053^**^ (0.021)	0.066^***^ (0.017)
School in village (1=Yes, 0=No)	0.150^**^ (0.063)	0.242^***^ (0.086)	0.248^***^ (0.073)
Max. parent’s education	0.213*** (0.025)	-	0.827^***^ (0.078)
Gender (1=Male, 0=Female)	-0.275^***^ (0.053)	-	-0.292^***^ (0.060)
Head’s gender (1=Male, 0=Female)	-0.243^***^ (0.076)	-0.287^**^ (0.114)	-0.149^**^ (0.071)
Age (Years)	0.810^***^ (0.008)	0.811*** (0.010)	0.812*** (0.006)
Age started school	-0.459^***^ (0.025)	-	-0.429^***^ (0.024)
Household size	-0.079^***^	-0.078^***^	-0.074^***^
	(0.014)	(0.017)	(0.013)
Observations	11992	11992	11992

*Notes*. Standard errors are in parentheses. Significance level: *p < .10, **p < .05, ***p < .01. Results are based on panel of children who have ever attended school and were 6 to 18 years old in 2008. Results are presented for key variables only, estimated model includes more variables. RE, FE, and HTIV stand for Random Effects, Fixed Effects, and Hausman-Taylor Instrumental Variable estimators, respectively.

Table 5 shows how the aggregated asset index affects highest grade completed. The aggregated index has the expected sign, suggesting wealth has positive effects on children’s education. The positive coefficient on consumption expenditure, a proxy for household income, also suggests positive income effects. Among other controls, both having educated parents and access to a school in the village help children reach higher grades; the effects on child education of a 5 percent increase in total expenditure and an increase in parental education by one more level (such as primary to secondary school) are identical. Educated parents may expect a larger return from sending children to school, so they may not consider the opportunity cost of schooling for their children to be high. Similarly, children who live near a school may both attend school and occasionally take part in farm-household activities. This would lead to the positive effect for ‘school in village’ even if the child has to work in agriculture. After controlling for endogeneity, the effect of parental education on children’s highest grade completed becomes more than quadruple the result found by the random effects model. This implies the potential endogeneity of parental education and shows the importance of preferring the HTIV method to the fixed effects model.

Interestingly, having a male head of household adversely affects children’s grade level, but girls are more likely to reach higher grades than boys. This is consistent with evidence from other developing countries that boys are more likely than girls to forgo school for agricultural activities because girls usually take care of household and kitchen activities (Akresh et al., 2013; Burke and Beegle, 2004). The level of education increases with age but children who start school late hurt their chances of reaching higher grades. Finally, household size has a smaller but significant negative effect on child education, suggesting that the larger the household, the less educated the child.

Table 6 disaggregates assets into household durables, agricultural assets, and housing quality assets. Although the Table 5 results suggest that assets uniformly contribute to child education through positive wealth effects, it appears from Table 6 that different types of assets have differential effects. Household durables and housing quality characteristics have the expected positive effects but agricultural assets have negative effects on highest grade completed. As agricultural assets include farm tools and equipment, land, and livestock, owning more agricultural assets may raise the opportunity cost of schooling and heighten demand for child labor, which contributes to school dropout. However, the adverse effect of agricultural assets is more than offset by household durables and good housing characteristics, which both have larger positive effects than agricultural assets. The estimated effects of other variables, such as access to a school in the village, are qualitatively identical to the Table 5 results discussed.

The evidence of the negative effects of agricultural assets on grade level completed is particularly striking because it challenges the traditional view that wealth has a positive effect on education. Agricultural (or any productive) assets are a form of wealth, but they may behave differently than durable assets and housing quality assets in that productive assets require that labor and other input costs be operational. Ownership of agricultural assets may indicate wealth acquisition but it may raise the opportunity cost of both schooling and demand for child labor, especially for agrarian households that have little or no access to other labor markets. From the evidence, an undifferentiated view of assets is misleading. Because ownership of agricultural assets raises the likelihood of child labor in own-farm activities (Appendix Table A2), presumably the opportunity cost of schooling rises with agricultural assets through an effect on child labor for farming.

That different assets have differential effects and that agricultural assets increase child labor in agriculture is so striking a result for policy makers and planners that it deserves further exploration. In Table 7, we estimate our preferred HTIV model for various subsamples to identify mechanisms that may be behind the differential effects of different types of assets. We estimate the model for eight subsamples — rural, urban, crop producers, livestock keepers, boys, girls, poor, and nonpoor — and find that different types of assets have differential effects among rural children and children from crop producers. Although in both cases the aggregated asset index has positive effects on child education, we find no evidence of asset-specific effects on educational outcomes of urban children and children from livestock producers. The results for boys vs. girls and poor vs. nonpoor subsamples are not discussed here, but we find no evidence of differential effects in these cases. This indicates that while positive wealth effects on child education are consistent in various scenarios, different types of assets have differential effects mostly for rural children and children from grain crop farmers. The results make perfect sense in that the opportunity cost of schooling may not go up with agricultural assets regardless of wealth status if the household is not farming. In rural areas, there are few if any labor markets and most surveyed households are active in agrarian settings, where more agricultural assets mean a higher opportunity cost for schooling.

#### 4.2.2 Effects of assets on test performance

The empirical results indicate that agricultural assets do have negative effects on highest grade completed, and these stem from the fact that child labor is largely used in agriculture and most agricultural assets complement child labor. However, although ‘highest grade completed’ is a valid measure of school enrollment and grade completion, it does not account for student effort and performance (nor school quality). To this end, we use the PSLE ratio to examine the effects of assets on how school-age children perform on the primary school leaving exam (Table 8) and the FIVE ratio to assess the effects of assets on how adolescents perform on leaving form IV (Table 9).

**Table 7:** Effects of different assets on highest grade completed of children ages 6 to 18, under various scenarios

	Model: HTIV			
	Rural	Urban	Grain crop farmers	Livestock keepers
Log(Total expenditure)	0.152^***^ (0.040)	0.258^***^ (0.071)	0.221*** (0.040)	0.191*** (0.044)
Household durable index	0.068^***^ (0.023)	0.021 (0.021)	0.057^***^ (0.022)	0.081^***^ (0.025)
Agricultural asset index	-0.026^***^ (0.008)	-0.007 (0.005)	-0.012* (0.007)	0.002 (0.008)
Housing quality index	0.123*** (0.024)	0.010 (0.028)	0.102*** (0.023)	0.109^***^ (0.026)
School in village (1=Yes, 0=No)	0.241^*^ (0.134)	0.074 (0.091)	0.314^***^ (0.101)	0.327^***^ (0.119)
Max. parent’s education	0.581^***^ (0.139)	0.470^**^ (0.192)	0.837^***^ (0.133)	0.759^***^ (0.145)
Gender (1=Male, 0=Female)	-0.313*** (0.065)	-0.185^*^ (0.102)	-0.289^***^ (0.065)	-0.298^***^ (0.070)
Head’s gender (1=Male, 0=Female)	-0.224^***^ (0.086)	-0.065 (0.128)	-0.144^*^ (0.085)	-0.075 (0.096)
Age (years)	0.764^***^ (0.007)	0.902^***^ (0.012)	0.782^***^ (0.007)	0.792^***^ (0.008)
Age started school	-0.420^***^ (0.031)	-0.437^***^ (0.054)	-0.422^***^ (0.030)	-0.405^***^ (0.033)
Household size	-0.073^***^ (0.016)	-0.084^***^ (0.024)	-0.078^***^ (0.016)	-0.068^***^ (0.016)
*Observations*	8095	3897	8796	7217

*Notes*. Standard errors are in parentheses. Significance level: *p* < .10, *p* < .05, *p* < .01. Results are based on panel of children who have ever attended school and were 6 to 18 years old in 2008. Results are presented for key variables only, estimated model includes more variables. HTIV stands for Hausman-Taylor Instrumental Variable estimator.

**Table 8:** Effect of different assets on PSLE performance of children ages 6 to 18

	Dep. variable: PSLE pass ratio		
	RE	FE	HTIV
Log(Total expenditure)	0.051^***^ (0.007)	0.035^***^ (0.009)	0.040^***^ (0.008)
Household durable index	0.012*** (0.003)	0.008^*^ (0.005)	0.010*** (0.004)
Agri. asset index	0.001^*^ (0.001)	0.000 (0.001)	-0.000 (0.002)
Housing quality index	0.018*** (0.003)	0.003 (0.006)	0.010^*^ (0.005)
School in village	0.007 (0.009)	0.012 (0.012)	0.011 (0.009)
Max parent’s education	0.006 (0.005)	-	0.073^***^ (0.019)
Head: age	0.003^***^ (0.000)	0.004^***^ (0.001)	0.005^***^ (0.001)
Head: Gender (1=male, 0=female)	-0.036^***^ (0.013)	-0.075^***^ (0.025)	-0.033^**^ (0.014)
Household size	-0.024^***^ (0.002)	0.002 (0.004)	-0.021*** (0.003)
Observations	6029	6029	6029

*Notes*. Standard errors are in parentheses. Significance level: *p < .10, **p < .05, ***p < .01. As the dependent variable is at the household level, no individual characteristics are included in the model. Results are presented for key variables only, estimated model includes more variables. RE, FE, and HTIV stand for Random Effects, Fixed Effects and Hausman-Taylor Instrumental Variable estimators, respectively and Primary school leaving exam (PSLE) is a national level examination administered after 7^th^ grade.

**Table 9:** Effect of different assets on FIVE performance, youth aged 18-24

	Dep. variable: FIVE pass ratio			
	RE	FE	HTIV
Log(Total expenditure)	0.036^***^ (0.006)	0.024^***^ (0.008)	0.031^***^ (0.008)
Household durable index	0.018*** (0.003)	0.004 (0.004)	0.009^***^ (0.003)
Agri. asset index	-0.000 (0.001)	-0.001 (0.002)	-0.002 (0.001)
Housing quality index	0.019*** (0.003)	0.009^*^ (0.006)	0.015*** (0.005)
School in village	0.008 (0.009)	0.007 (0.011)	0.010 (0.008)
Max parent’s education	0.011*** (0.004)	-	0.077^***^ (0.019)
Head: age	0.001^***^ (0.000)	0.001 (0.001)	0.003^***^ (0.001)
Head: Gender (1=male, 0=female)	-0.029^**^ (0.013)	-0.033 (0.035)	-0.018 (0.013)
Household size	-0.010*** (0.001)	-0.000 (0.003)	-0.006^***^ (0.002)
Observations	5219	5219	5219

*Notes*. Standard errors are in parentheses. Significance level: *p* < .10, *p* < .05, *p* < .01. As the dependent variable is at the household level, no individual characteristics are included in the model. Results are presented for key variables only, estimated model includes more variables. RE, FE, and HTIV stand for Random Effects, Fixed Effects, and Hausman-Taylor Instrumental Variable estimators, respectively and Form IV exam (FIVE) is a national level examination administered after 11^th^ grade.

Still using equation (8), we estimate the same two model specifications, one with an aggregated asset index and another with the indexes disaggregated, but this analysis is carried out at the household

level. While the variables of interest are still the same, in the new control covariates, household controls replace all individual controls.^8^ Results from the first specifications are not presented here but, as expected, we find positive wealth effects on children’s performance in both the PSLE and the FIVE (Appendix Tables A3 and A4).

**Table A3:** Effect of asset ownership on primary school leaving exam performance

	Dep. variable: PSLE pass ratio		
	RE	FE	HTIV
Log(Total expenditure)	0.056^***^ (0.007)	0.036*** (0.009)	0.041^***^ (0.008)
Asset index	0.019^***^ (0.002)	0.011** (0.005)	0.016*** (0.003)
School in village	0.008 (0.009)	0.013 (0.011)	0.014 (0.009)
Max parent’s education	0.006 (0.005)	-	0.063^***^ (0.019)
Head: age	0.003^***^ (0.000)	0.004*** (0.001)	0.005^***^ (0.001)
Head: Gender (1=male, 0=female)	-0.032^**^ (0.013)	-0.072^***^ (0.025)	-0.030^**^ (0.014)
Household size	-0.023^**^ (0.003)	0.002 (0.004)	-0.020^***^ (0.003)
*Observations*	6029	6029	6029

*Notes*. Standard errors are in parentheses. Significance level: *p* < .10, *p* < .05, *p* < .01. As the dependent variable is at the household level, no individual characteristics are included in the model. Results are presented for key variables only, estimated model includes more variables. RE, FE, and HTIV stand for Random Effects, Fixed Effects, and Hausman-Taylor Instrumental Variable estimators, respectively and Primary school leaving exam (PSLE) is a national level examination administered after 7^th^ grade.

**Table A4:** Effect of asset ownership on FIVE performance

	Dep. variable: FIVE pass ratio		
	RE	FE	HTIV
Log(Total expenditure)	0.042^***^ (0.006)	0.025^***^ (0.007)	0.029^***^ (0.008)
Asset index	0.025^***^ (0.002)	0.008^*^ (0.005)	0.018*** (0.003)
School in village	0.010 (0.009)	0.007 (0.011)	0.013 (0.008)
Max parent’s education	0.011*** (0.004)	-	0.078^***^ (0.018)
Head: age	0.002^***^ (0.000)	0.001 (0.001)	0.004^***^ (0.001)
Head: Gender (1=male, 0=female)	-0.022^*^ (0.013)	-0.032 (0.035)	-0.014 (0.013)
Household size	-0.009^***^ (0.001)	0.000 (0.003)	-0.005^**^ (0.002)
*Observations*	5219	5219	5219

*Notes*. Standard errors are in parentheses. Significance level: *p < .10, **p < .05, ***p < .01. As the dependent variable is at the household level, no individual characteristics are included in the model. Results are presented for key variables only, estimated model includes more variables. RE, FE, and HTIV stand for Random Effects, Fixed Effects, and Hausman-Taylor Instrumental Variable estimators, respectively and Form IV exam (FIVE) is a national level examination administered after 11^th^ grade.

Table 8 presents the estimated effects of asset holdings on the PLSE ratio, the proportion of school-age children passing the PSLE exam. Results from the second specification, where the asset index is disaggregated into three subindexes, show that the positive effect of wealth on PSLE performance mainly comes from household durables and housing quality assets. However, unlike ‘highest grade completed’, PSLE performance is not affected at all by agricultural assets. Similar results also hold for the FIVE; the aggregated wealth index has a strong positive effect on the FIVE ratio that stems from the effects of household durables and housing quality index, but agricultural assets have no effect on adolescent performance on the FIVE (Table 9). This suggests that the effect of agricultural assets is not the same for children from the same household and may depend on each child’s ability.

A candidate (untested) hypothesis for the heterogeneous effects across children is that children doing well in school may not be as affected by agricultural assets because the returns parents expect from sending more able children to school may be higher than the expected return from investing in the education of less able children (Akresh et al., 2012). As a consequence, children who were not doing well in school may have had no opportunity to take the tests because they may have been taken out of school for farm activities. Since having more agricultural assets may be an incentive for parents to take children performing poorly out of school, agricultural assets adversely affect the highest grade completed but do not affect test performance because children who take the tests are mostly high- ability students.

Among other variables, household consumption expenditure has a strongly positive effect on PSLE and FIVE ratios, suggesting that income has a positive effect on child educational outcomes. Similarly, maximum parental education contributes to enhanced performances in both tests, but unlike the effects on ‘highest grade completed’, having a school in the village has no effect on children’s performance on either test. One possible implication is that students who are doing well and still in school may find it worthwhile to travel farther to a nearby community for schooling, but students who are not doing well may drop out when school is farther away.

#### 4.2.3 Robustness check

We run several alternative specifications for all three outcome variables — highest grade completed, the PSLE ratio, and the FIVE ratio — and the results are reasonably consistent with the findings from the main specifications. To examine whether consumption is absorbing the effects of assets on child education (through “income effects”), we exclude the consumption expenditure variable from the model specification and estimate the effects of different types of assets. The result is that excluding consumption expenditure slightly attenuates the negative effects of agricultural assets (the coefficient estimate decreases from —0.013 to —0.014) but amplifies the positive effects of both household durables (0.026 to 0.036) and housing quality (0.066 to 0.073). A similar pattern holds for both PSLE and FIVE ratios: no significant change in the effects of agricultural assets but effects of other assets that are more positive. Our choice to include consumption in our preferred specification (as a control for the overall well-being of the household) does appear to work slightly against our story. To address concerns about the possible endogeneity of the household size variable, we run our preferred model specification (HTIV with the expenditure variable included), specifying household size as a time-varying endogenous variable. The results are consistent with the main results: the negative effect of household size remains the same, leaving no evidence for positive effects of household size through economies of scale effects.

## 5 Conclusion

There is considerable empirical evidence that household wealth helps improve child education (Deng et al. 2014; Chowa et al. 2013; Huang 2013; Elliott et al. 2011; Kim and Sherraden 2011; Shanks 2007; Zhan and Sherraden 2003; Conley 2001). Despite the positive effect of household wealth, there is very little empirical evidence on how different components of wealth (different assets) contribute to child education. In this paper, we applied a simple theoretical model that maps a conceptual pathway for different types of assets to have differential effects on child education. Our model predicts that under the assumption of perfect labor markets, an increase in assets contributes to improved child education indicators; but assuming imperfect labor markets implies that the effect of assets is ambiguous, depending on type of assets and other conditions. Under the assumption of incomplete labor markets, our empirical results confirm the theoretical findings and reveal that different assets have differential effects on child education, presumably through child labor.

We have shown that agricultural assets have adverse effects on the highest grade completed but no effect on children’s test performance in our data. This implies that agricultural assets may increase the opportunity cost of schooling but the increment may not be homogenous among all children in the same household. For children who are doing well in school, the opportunity cost of schooling is warranted because the return from their education is higher than that expected from educating other children. As child schooling largely depends on parental decision about when and which child to send to school, parents may choose to take the less able children out of school and invest more in educating the more able children. This leads to agricultural assets having a negative effect on grade completed or school enrollment but no effect on school performance. That agricultural assets have negative effects on child education because they increase the opportunity cost of schooling is substantiated with the evidence of larger negative effects of agricultural assets for children working in household agricultural activities. Our finding that the negative effects of agricultural assets are amplified for rural children and children of crop producers also reinforces the inference that the negative effect of agricultural assets operates through child agricultural labor.

Unlike agricultural assets, household durables and housing quality are not complements to child labor and are therefore unlikely to increase the opportunity cost of schooling. Indeed, these asset indices have positive effects on both grade completed and exam performance. Household durables are part of household wealth, and these may contribute to better education for children through standard wealth effects. These assets may also enhance economic security and reduce economic stress for parents, which usually leads to better child education through good parenting. Housing quality may also work simply through wealth effects, but some dimensions of this may have more direct effects: electricity makes studying more efficient, and access to safe water and good sanitation facilities may improve school performance by improving child health.

Even though assets overall serve as a good predictor of child educational performance, interventions to enhance agricultural assets may not be favorable for education outcomes in some contexts. If child education is an intended goal, transferring agricultural assets may not yield the desired result. Nonetheless, there may be ways to increase agricultural asset holdings without compromising educational outcomes. Since the negative effect emerges through child labor in agriculture, making an asset-based intervention policy conditional on school attendance, or ‘no child labor in agriculture’, may enhance household welfare without hurting child education—although applying such a policy may be extremely difficult. Another implication of our findings is that transferring agricultural assets to parents in combination with awareness training or adult education for them, or establishing a public school in the target community may mitigate the potential adverse effects of agricultural assets on child education.

Because programs that help accumulate household durables or improve housing quality contribute to child education, they could be incorporated into policy interventions for improving both household welfare and child education. Although such policy interventions are rare, our empirical findings suggest that interventions that combine transfers of agricultural with household durable or housing quality assets may both heighten household socioeconomic status and temper the possible negative effect of agricultural assets. Since we control for household income, our findings should hold regardless of household income. One caveat is that this study does not consider the threshold of income or asset holdings above which change in the value of assets held may have no effect because demand for child education is inelastic to the opportunity cost of schooling.

The main lesson from this study is that assets are an important element of social policies designed to improve both household and individual welfare. The conventional practice of considering all the assets households possesses as an aggregate measure of household wealth may be misleading because different types of assets have differential effects on child education, something that may also be true for other outcomes. The evidence that, even after controlling for household income, having assets has a significant positive effect on child education but the effect differs by type of assets, is a novel finding that warrants further exploration. If similar findings hold for other countries and contexts, that should help researchers and policy makers to design interventions that promote accumulation of assets in a more strategic way.

## Footnotes

1. Their experiment had six elements, including asset transfer, training, and short-run support, but they consider the asset transfer to be the core component of the program. They found that the positive effects continued three years after receipt of the asset transfer, and the positive effects are seen in all six countries where the experiment was carried out (Ethiopia, Ghana, Honduras, India, Pakistan, and Peru).

2. For a survey of the literature, see Elliott et al. (2011).

3. The Tanzania NPS is part of the LSMS-ISA program which aims to marry complex consumption-based household surveys with plot-crop detailed agricultural surveys. The Tanzania NPS data, along with details on the sample and instrument design, are publicly available.

4. The second partial derivative of utility with respect to consumption being negative simply assumes that there are diminishing marginal returns to increasing consumption. The motivation for assuming that uu < 0 is that because time is constrained, as labor increases, time for schooling decreases and as it approaches zero, the disutility from working increases more rapidly.

5. To the best of our knowledge, no previous studies have introduced an education production function into an agricultural household model. Doing so complicates the model but the added complexity help us understand the potential effects of assets and tools that are not used in agricultural production and may have a direct impact on child education. We argue that models with child education functions are more realistic because most agricultural households face production, consumption, and child education decisions simultaneously.

6. This seems to be a reasonable assumption because the marginal rate of substitution between child labor and consumption may decrease with consumption, i.e., $z_{c}=\frac{\delta }{\delta }\frac{ul}{u_{c}}=\frac{ul_{c}u_{c}}{u_{c}^{2}}<0$ because *u_c_* > 0, u_*lc*_ < 0 and *u_l_,u_cc_* < 0, by assumption. Similarly, *Z_l_* < 0.

7. In practice, HTIV can be estimated using the STATA in-built command ‘Xthtaylor’. We use the Xthtaylor command, specifying asset indexes and parental education as endogenous variables. Conceptually, first equation (7) is estimated with the fixed effects model saving the residual. The residual is used to run a regression on z_1i_ and z_2i_ by using *x*_1*it*_ and *z*_1i_ as instruments. All variables in the model are then transformed by using the estimated variance from the residual regression. The transformed model is estimated by using $x_{1it}-{\bar{x}}_{1i},\;x_{2it}-{\bar{x}}_{2i},\;z_{1i}\;\mathrm{and}\;{\bar{x}}_{1i}$ as instruments.

8. The new set of control variables are log(total expenditure), education of head, age of head, sex of head, marital status of head, household size, number of children, and binary indicators for residence in the mainland or Zanzibar and economic shock in the last 12 months.
